# Plasticity of Fine-Root Traits Under Long-Term Irrigation of a Water-Limited Scots Pine Forest

**DOI:** 10.3389/fpls.2019.00701

**Published:** 2019-06-04

**Authors:** Ivano Brunner, Claude Herzog, Lucía Galiano, Arthur Gessler

**Affiliations:** ^1^Forest Soils and Biogeochemistry, Swiss Federal Institute for Forest, Snow and Landscape Research WSL, Birmensdorf, Switzerland; ^2^Forest Dynamics, Swiss Federal Institute for Forest, Snow and Landscape Research WSL, Birmensdorf, Switzerland; ^3^Centre for Research on Ecology and Forestry Applications, Barcelona, Spain; ^4^Department of Animal, Plant Biology and Ecology, Universitat Autònoma de Barcelona, Barcelona, Spain

**Keywords:** architectural traits, biomass, drought, dynamic traits, ingrowth cores, morphological traits, production, soil coring

## Abstract

Trait-based approaches are increasingly used to investigate plant strategies for resource acquisition, growth, or competition between individual organisms or across species. However, the characterization of responses to environmental stimuli by fine-root systems of trees at the trait level is rather limited, particularly regarding the timing and degree of plasticity of the traits involved. These aspects become especially relevant under current climate-driven shifts in environmental conditions. In the present study, we examined the responses of the fine roots of Scots pines to increased soil water availability from long-term irrigation starting in the year 2003. The Scots pine forest is situated in a water-limited region in the central European Alps where increased tree mortality has been observed over the last two decades. The fine-root traits investigated include root system traits, root dynamic traits, architectural traits, and morphological traits. A first survey of fine-root traits in 2005 using ingrowth cores did not reveal any trait-based responses resulting from the irrigation treatment over a three-year period. Fine-root biomass, as periodically recorded by coring the topsoil from 2003 to 2016, showed a significant increase compared to the non-irrigated controls between three and nine years after the start of treatment. Overall, a maximum biomass increase due to the irrigation treatment was recorded in 2016 with about 80% higher biomass compared to controls. The analysis of fine-root traits revealed that irrigation significantly increased biomass, length, and production, but did not alter morphological and architectural traits, such as diameter, frequency of tips, specific root length (SRL), and root tissue density (RTD). In contrast, clear significant differences were found for all traits except for length when comparing the two root sampling methods, namely, ingrowth cores and soil coring. However, there were no interactions between the irrigation treatment and the sampling methods used and, therefore, the methods used did not affect the documented patterns, just the actual measured trait values.

## Introduction

Trait-based approaches are increasingly being used to investigate plant strategies for resource acquisition, growth, and competition, as well as for plant impacts on ecosystem processes (McCormack et al., 2017). However, most efforts have thus far focused on aboveground plant traits, while belowground traits and strategies still remain under-researched. To fill this gap, a Fine-Root Ecology Database (FRED^1^) was created exclusively for fine-root traits and which now includes over 70,000 observations (Iversen et al., 2017).

Ideally, the range of a trait’s response to environmental stimuli or plant-intrinsic factors should be known. Regrettably, this knowledge is still limited for belowground traits (Shipley et al., 2016). Part of the problem may stem from the inconsistency and inaccuracy of belowground measurements (Freschet et al., 2015; McCormack et al., 2017).

The variability of a trait in response to environmental changes, either across environmental gradients or in experimental treatments, is called “phenotypic plasticity” (Callaway et al., 2003; Iversen et al., 2017). The capacity of organisms to alter their phenotype under changing conditions is widely recognized as an important mechanism to avoid migration or extinction (Valladares et al., 2014). The term phenotypic plasticity is currently applied in a broad sense, and is used to describe the phenotypic responses of organisms to environmental change, e.g., acclimation (equivalent to acclimatization) (Kelly et al., 2012). One aboveground example is the particular plasticity of the leaves of tropical trees, which grow differently depending on light conditions. In the light, leaves are thicker, whereas in the shade they tend to be thinner (Rozendaal et al., 2006). Weemstra et al. (2017) and Zadworny et al. (2017) have recently shown that phenotypic plasticity occurs in tree roots, where physical and chemical soil parameters are the influencing factors instead of light.

Plasticity is understood as an adaptive mechanism that allows plants to optimally respond to environmental heterogeneity (Palacio-Lopez et al., 2015). The phenotypic shift can either be the result of a genetic differentiation of populations to become locally adapted, or of the phenotypic plasticity of individuals expressing the optimal phenotype for the corresponding environment via physiological and molecular regulatory mechanism (Palacio-Lopez et al., 2015). However, the ability of an organism to express plasticity within a given trait is most likely mediated at the molecular level (Nicotra et al., 2010).

Here, we examine the response of Scots pine (*Pinus sylvestris* L.) fine-root traits to precipitation changes using a unique long-term irrigation experiment in a water-limited pine forest ecosystem in the central European Alps (Dobbertin et al., 2010; Hartmann et al., 2017). The irrigation experiment was established in the Pfyn forest in 2003 to better understand how forest ecosystems respond to water limitation (Dobbertin et al., 2010). The Pfyn forest represents the largest continuous forest of Scots pine in Switzerland, and is located in the dry Rhone Valley. Large-scale Scots pine forests in the transition zone between continental and Mediterranean climates are characteristic landscape elements in dry and warm inner Alpine valleys in the Central Alps (Rigling et al., 2013). Increasing Scots pine mortality has been recorded for several decades, with a dieback of up to 50% in particularly water-limited stands in the Swiss Rhone Valley since 1995, as well as in other valleys in the central Alps in Italy and Austria (Rebetez and Dobbertin, 2004; Vacchiano et al., 2012). High mortality of Scots pine on long-term monitoring sites in the Swiss Rhone valley, with up to 20% of the trees in a stand dying in a given year, was observed in 1999, 2004, and 2017. At the Pfynwald site, cumulated mortality between 2003 (when the irrigation treatment started) and 2016 was about 16% in the control plots and about 9% in the irrigated plots. Although average annual precipitation has remained constant in recent decades, there is evidence that climate warming has increased evaporation rates, and that water has become the main factor limiting growth and reducing stress resilience in trees (Rigling et al., 2013). It was therefore hypothesized that reducing stress from water limitation using irrigation could improve tree vitality and reduce mortality. Indeed, after 3–9 years of irrigation, trees showed increased leaf area and increased fine-root biomass (Dobbertin et al., 2010; Herzog et al., 2014). Furthermore, irrigation was found to cause significant shifts in plant community composition and increased vegetation cover (Herzog et al., 2014). In response to the doubling of precipitation over the decade-long experimental period, the monthly mean volumetric water content in the top soil increased significantly, from 28% in the controls to 34% in the irrigated plots (Hartmann et al., 2017).

In the present study, our research question was to determine whether the irrigation treatment (i.e., dry controls vs. irrigation) would affect the recorded fine-root traits. The second research question was to determine whether the method of recording roots (soil cores vs. ingrowth cores) under the irrigation treatment would influence the traits. It was the aim of the study to investigate the fine-root traits of several trait categories (McCormack et al., 2017), such as “root system,” “morphology,” “architecture,” and “root dynamics.”

## Materials and Methods

### Study Site and Experimental Setup

The study site is located in the Pfynwald forest situated in the Rhone Valley in Switzerland (46°180 N, 07°370 E, 615 m.a.s.l.), in a Scots pine forest containing trees of about 100 years of age, a stand density of about 730 stems ha^–1^, and with occasional interspersed pubescent oak (*Quercus pubescens* Willd) (Brunner et al., 2009). The mean yearly precipitation of the nearby climate station Sion is 603 mm, with a mean annual temperature 10.2°C (1980–2010; MeteoSchweiz, 2018). Over the last three decades, precipitation has dropped toward 90% of the long-term average and the temperature has risen by more than 1°C (1980–2010; MeteoSchweiz, 2018). For the long-term irrigation treatment, a 1.23-ha study area containing about 1,100 tree individuals, was subdivided into eight plots of 25 × 40 m (1,000 m^2^) with 5 m buffer areas between and around each plot. The plots were aligned side by side along a channel fed by the Rhone River, from where water was taken to irrigate four randomly selected plots (hereafter referred to as “irrigated”). Four plots were left untreated as control plots (hereafter “dry”). Irrigation started for the first time in spring 2003. The irrigation system was activated on rainless nights during the vegetation period (May-October), doubling the annual rainfall amount. Volumetric soil water content was monitored hourly using time-domain reflectometry (Tektronix 1502B cable tester, Beaverton, OR) at soil depth of 10 cm at four different locations in irrigated and dry plots. The mean volumetric water content in the soil significantly increased from 28% in the dry plots to 34% in the irrigated plot (Herzog et al., 2014). In 2013, irrigation was stopped in about a third of the area of each of the irrigated plots to track the return to a ‘dry’ forest. More details on the study site and experimental setup are described in Brunner et al. (2009), Herzog et al. (2014), and Hartmann et al. (2017), and data on long-term growth as well as on relative leaf areas of trees from the irrigated and control plots are given by Schönbeck et al. (2018).

### Soil Cores

Fine roots (<2 mm in diameter) were sampled from healthy sample trees with a soil-coring cylinder (diameter 4.5 cm) about 0.5–1 m away from the stems and down to the rocks of the subsoil to a depth of 8–12 cm. Two soil cores per tree were taken, and three trees per plot were sampled before irrigation treatment started and in April / May in 2003, 2004, 2005, 2012, 2014, and 2016 (Brunner et al., 2009; Herzog et al., 2014). The same trees were sampled throughout the duration of the experiment. Overall, twelve trees in the dry- and nine trees in the irrigation-treatment were included in the analysis. After sampling, the soil cores were packed in plastic bags, transported to the laboratory, and stored at a low temperature (in a cold room at 4°C) until they were analyzed. The soil cores were then washed in a sieve, the roots collected, and the fine roots of Scots pine sorted out by hand, dried at 60°C for 3 days, and weighed. In order to compare data over the 13-year period, the two samples per tree were averaged. Fine roots were calculated per cm^3^ of soil and then balanced for 0–10 cm depth and per m^2^ to obtain comparable data.

### Ingrowth Cores

A first series of ingrowth cores (glass-fiber-netting cylinders 11 cm in height, 5 cm in diameter, with a 5 mm mesh size) were set in April 2003 with the ingrowth cores inserted into the holes where soil core samples had been taken previously for the fine-root biomass (Brunner et al., 2009). The ingrowth cores were refilled with sieved topsoil from outside the plots. The ingrowth cores were then harvested with a large soil corer 8.5 cm in diameter after 2 years in May 2005. After harvest, the ingrowth cores were packed undisturbed in plastic bags, transported to the laboratory, and stored at 4°C until they were analyzed several days later. The ingrowth cores were then cut out with a knife, the core length of the samples recorded, the netting removed with scissors, the soils sieved, and the fine roots rinsed with tap water. The fine roots of the Scots pine were then sorted out by hand, and stored in tap water in a refrigerator until fine-root morphology and architecture was analyzed (Brunner et al., 2009).

A second series of ingrowth cores, using identical ingrowth cores as before, was installed in April 2014 and harvested in spring 2016. The ingrowth cores were installed in the same way as the first series, at a distance of about 0.5–1 m from the stems. However, trees other than those from the first series were probed, and three ingrowth cores per tree instead of two were installed. In total, six trees were selected from the dry treatment and six trees from the irrigated treatment.

In order to compare the datasets of the two series of ingrowth cores, the data were averaged per tree. If one of the ingrowth cores per sample tree remained without roots, then only those with roots were taken into consideration.

### Fine-Root Scanning and Analyses

The fine roots from the ingrowth campaign of 2005 and 2016, and from the soil-coring sampling in 2016 were scanned for morphological characteristics before drying and weighing. The scanned pictures were then analyzed using the WinRHIZO software package (version 4.1c, Regent Instruments Inc., Quebec, Canada) for morphological and architectural traits such as length, diameter, root volume, tips, and forks.

### Fine-Root Traits

The measured fine-root traits were root biomass density (g m^–2^), root length density (m m^–2^), mean diameter (mm), tip frequency (n cm^–1^), and fork frequency (n cm^–1^). The calculated fine-root traits were specific root length (SRL; m g^–1^) and root tissue density (RTD; g cm^–3^). Biomass and length were calculated per soil area and to a soil depth of 10 cm (topsoil), tips and forks to the root length, SRL to the fine-root biomass, and RTD to the fine-root volume (Brunner et al., 2009). The fine-root turnover rate (year^–1^) was calculated by dividing annual production by standing biomass (Gill and Jackson, 2000; Brunner et al., 2013) by using ingrowth core data for the annual production and soil-coring data for the standing biomass for the years 2005 and 2016. The method used to determine turnover rate, however, is only a rough estimate because of root pruning that occurs when ingrowth cores are installed (Hendricks et al., 2006). The lifespan of the fine roots (year) is equal to the inverse value of the turnover rate (Brunner et al., 2013).

### Statistical Analyses

The statistical analyses performed were one- and two-way analyses of variance (ANOVA), repeated-measures ANOVA, and simple linear regression with ANOVA using StatView software (Version 5.0, SAS Institute, Cary, NC). Repeated-measures ANOVA was applied when samples were taken consecutively from the same trees at different times. The significance of the differences between treatments was tested by pairwise comparison of the sample means using Fisher’s protected least significant difference at *P* < 0.05. All data were tested for normal distributions using the Kolmogorov-Smirnov Test of Normality^2^.

## Results

### Fine-Root Biomass

**FIGURE 1:**
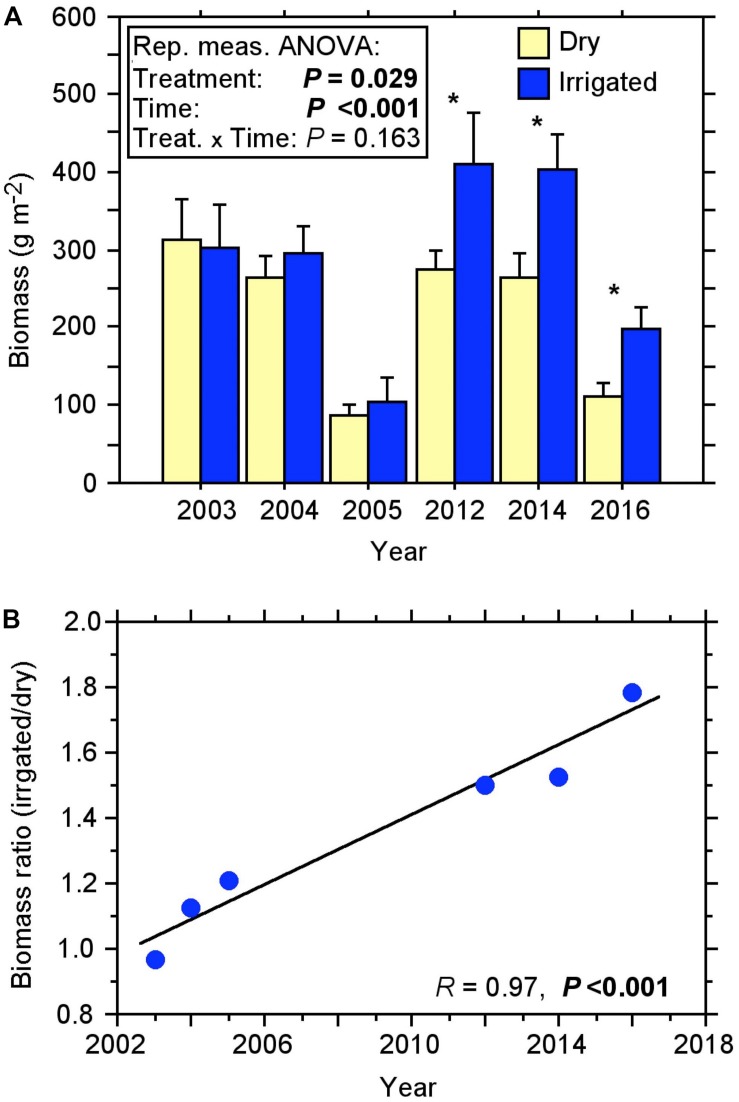
Fine-root biomass of the topsoil (0–10 cm) under dry and irrigated conditions of the Scots pine forest for the years 2003 to 2016 recorded by soil coring. **(A)** Course of biomass (±SE). Repeated measures ANOVA, significant effects (*P* < 0.05) are shown in bold; One-way ANOVA, significant effects (*P* < 0.05) are indicated with ^*^. **(B)** Relationship between the biomass ratio (irrigated/dry) and the sample years, a significant effect (*P* < 0.05) is shown in bold.

The fine-root biomass of the Scot pines, as recorded with soil coring over more than a decade, changed considerably over the duration of the experiment (Figure 1A). Under the dry (control) condition, biomass varied from 87 to 311 g m^–2^ in the first 10 cm of the soil. In the first 2 years after the beginning of irrigation treatment, no significant change in the fine-root biomass was measured. Only when fine-roots were recorded again after a gap of 7 years in 2012, did they show a significant difference, with a biomass increase of approximately 50% in the irrigated compared to the dry plots (Figure 1B). This difference between treatments increased steadily over the following years (2014, 2016) resulting in the fine-root biomass of irrigated plots being more than 50% higher than in controls, with a maximum biomass of 411 g m^–2^ in 2012.

Repeated measures ANOVA revealed that the biomass increase in the irrigation treatment was statistically significant over the 13-year period (Figure 1A). Considering the ratio of biomass from the irrigated plots to dry plots, it is evident that over the years there has been a steady increase in the biomass ratio with a high coefficient of determination *R*^2^ of 0.95 (Figure 1B). We found no indication for a slowdown or reversal of this increase over time.

### Fine-Root Production and Turnover Rate

Comparing the 2005 fine-root production with that of 2016 reveals that values in general can be highly plastic, ranging from 33 to 66 g m^–2^ year^–1^ (Table 1). The data also show that production in 2005 was not significantly affected by the irrigation treatment. In contrast, in 2016, fine-root production significantly increased by a factor of 2. However, the turnover rate in 2016 was only slightly influenced by the irrigation treatment (Table 1) because the biomass recorded through soil coring had also greatly increased due to irrigation (compare also Figure 1A). The lifespan of the fine-roots, as calculated from the turnover rates, was from 1.4 to 1.6 year in 2005, whereas in 2016 it was found to range from 3.0 to 3.3 year (Table 1).

**TABLE 1:** Biomass, production, turnover rate, and lifespan of fine roots in the years 2005 and 2016 of the Scots pine forest under dry and irrigated conditions.

**Fine-root traits**	**Year 2005**			**Year 2016**		
	**Dry**	**Irrigated**	***P***	**Dry**	**Irrigated**	***P***
Biomass (g m^–2^)	87.2	105.3	0.55	111.5	198.5	**0.01**
Production (g m^–2^ y^–1^)	54.9	53.2	0.89	33.8	66.1	**0.02**
Turnover (y^–1^)	0.73	0.61	–	0.30	0.33	–
Lifespan (year)	1.38	1.64	–	3.31	3.00	–

*Biomass corresponds to standing biomass estimated by soil coring, production corresponds to annual production estimated by ingrowth cores, turnover corresponds to turnover rate (calculated as production divided by biomass), lifespan corresponds to root age and is the inverse of turnover. Turnover and lifespan were calculated per treatment. One-way ANOVA; significant effects (*P* < 0.05) are shown in bold.*

### Fine-Root Traits

Analysis of ingrowth cores sampled in 2005 showed that the fine-root traits measured were not significantly influenced by the irrigation treatment after 2 years of growth (Table 2). However, when the traits were again measured in 2016 from ingrowth cores, significant increases were then recorded for the biomass, length, and frequency of forks, but not for the average diameter or frequency of root tips, SRL, or RTD (Table 2). The strongest effects due to irrigation were recorded for root biomass, which grew 1.9-fold, and for overall root length, which increased 1.8-fold. When the 2016 soil coring samples were evaluated, only root biomass and overall root length increased significantly, 1.6 and 1.8-fold, respectively. This is slightly lower compared to the ingrowth cores (Table 2). Overall, fine-root architectural traits, such as the frequency of tips and forks, and morphological traits, such as average diameter, SRL and RTD, were not significantly influenced by the irrigation treatment (with one exception, namely, the frequency of forks in ingrowth cores in 2016). However, traits varied considerably across survey years and the method applied. Specifically, average diameters ranged from 0.7 to 1.1 mm, root tips from 1.1 to 3.4 cm^–1^, fork frequency from 1.1 to 5.9 cm^–1^, that of SRL from 7.2 to 13.3 m g^–1^, and that of RTD from 0.17 to 0.32 g cm^–3^ (Table 2).

**TABLE 2:** Comparison of fine-root traits of roots from ingrowth cores from 2005 and 2016 after 2 years of growth, and of roots from the soil-coring sample from 2016 of the Scots pine forest under dry and irrigated conditions.

**Fine-root traits**	**Ingrowth cores (2005)**			**Ingrowth cores (2016)**			**Soil cores (2016)**		
	**Dry**	**Irrigated**	***P***	**Dry**	**Irrigated**	***P***	**Dry**	**Irrigated**	***P***
Biomass (g m^–2^)	109.9	106.3	0.89	67.6	132.2	**0.02**	111.9	198.5	**0.01**
Length (m m^–2^)	38.9	59.9	0.14	87.3	157.0	**0.01**	100.2	155.4	**0.049**
Diameter (mm)	1.10	1.05	0.63	0.73	0.70	0.37	0.91	0.93	0.55
Tips (n cm^–1^)	1.12	1.10	0.81	3.21	3.43	0.26	2.39	2.40	0.85
Forks (n cm^–1^)	1.04	1.17	0.60	4.72	5.91	**0.01**	3.42	3.74	0.23
SRL (m g^–1^)	8.11	7.19	0.77	12.5	13.3	0.61	9.79	8.56	0.33
RTD (g cm^–3^)	0.32	0.37	0.64	0.20	0.22	0.19	0.17	0.18	0.50

*One-way ANOVA; significant effects (*P* < 0.05) are indicated in bold. SRL, specific root length; RTD, root tissue density.*

### Soil Cores vs. Ingrowth Cores

When fine-root traits from both ingrowth cores collected after two-year of growth and soil cores were sampled and analyzed in 2016, significant differences between irrigated and control samples were obtained for biomass, length and forks, but not for other fine-root traits (Table 3). A significant effect for the sampling method (ingrowth cores vs. soil cores) was observed for almost all fine-root traits, except for root length (Table 3). The greatest significant differences were recorded for fine-root tips, forks, average diameter, and SRL. Interactions between the two factors, irrigation treatment and fine-root sampling method were, however, not detected (Table 3).

**TABLE 3:** Results of two-way ANOVA testing the effect of the irrigation treatments (dry vs. irrigated) and the fine-root sampling methods (ingrowth cores vs. soil cores) on fine-root traits in 2016.

**Fine-root traits**	**Treatment**			**Method**			**Treatment × Method**		
	***dF***	***F***	***P***	***dF***	***F***	***P***	***dF***	***F***	***P***
Biomass (g m^−2^)	1	11.9	**0.002**	1	6.36	**0.02**	1	0.25	0.62
Length (m m^−2^)	1	10.2	**0.003**	1	0.09	0.77	1	0.14	0.71
Diameter (mm)	1	0.12	0.73	1	67.5	**<0.001**	1	1.21	0.28
Tips (n cm^−1^)	1	1.82	0.19	1	110.6	**<0.001**	1	1.34	0.26
Forks (n cm^−1^)	1	12.1	**0.002**	1	63.4	**<0.001**	1	4.06	0.05
SRL (m g^−1^)	1	0.05	0.83	1	14.4	**<0.001**	1	1.06	0.31
RTD (g cm^−3^)	1	1.70	0.20	1	7.85	**0.01**	1	0.15	0.71

*Significant effects (P < 0.05) are shown in bold. SRL, specific root length; RTD, root tissue density.*

## Discussion

Several attributes in the ecosystem of the experimentally irrigated pine forest changed due to the prolonged treatment over more than a decade. Herb and moss coverage increased significantly, as did crown cover (from 57 to 71%), tree biomass (from 6.8 to 7.9 kg m^–2^), and litter-fall (from 0.31 to 0.46 kg m^–2^ year^–1^) (Herzog et al., 2014; Hartmann et al., 2017). The increase in fine-root biomass only became apparent 9 years after the irrigation experiment began (Herzog et al., 2014). Although the exact time-point of a significant increase in fine-root biomass was not recorded, root biomass acclimation had started before year nine as indicated by the slight but not significant irrigation effects in 2004 and 2005. It also appears that the ratio of irrigated to dry treatment biomass was still increasing, rising from 1.5 in 2012 to 1.8 in 2016. This indicates that, on the one hand, a saturation of the fine-root biomass in the topsoil had not yet been reached, and, on the other hand, the density of fine-roots is strongly dependent on water availability. Thus, water rather than nutrients (e.g., nitrogen) seems to be the primarily limiting factor for fine-root abundance in this Scots pine forest. Long-term irrigation did not affect the carbon-to-nitrogen ratio, although the fine-root biomass increased by approximately 50% (Herzog et al., 2014). Thus, higher water availability did not mobilize more nutrients from the topsoil, which, in fact, could also promote fine-root growth (e.g., Yuan and Chen, 2012). An additional indication for the water limitation is the decrease in the $\delta^{13}$C-values in fine-roots from −26 to −27‰ (Herzog et al., 2014), and in trunk tree rings, from −22 to −24‰ (Timofeeva et al., 2017) in the irrigation treatment. These lower values are most likely a result of higher stomatal conductance, leading to higher leaf internal CO_2_ concentrations and subsequent higher photosynthetic carbon isotope fractionation (Farquhar et al., 1989).

Only recently, Solly et al. (2018) unraveled the real age of fine roots by counting annual rings, and discovered a mean age (=lifespan) of fine roots of 1 to 2 year (including the fine roots of the Scots pines in the Pfyn forest). Thus, the short lifespan of the fine roots explains the strong fluctuation between 2004 and 2005 and between 2014 and 2016, when fine-root biomass was reduced by more than half. This decrease was most likely due to the heat waves and summer droughts in 2003 and 2015, respectively (Ciais et al., 2005; Dietrich et al., 2018), which negatively affected fine-root biomass build-up with a delay of one to two years, which is when reduced storage reserves came to bear.

The fine-root turnover rate, estimated by dividing annual production by standing biomass, revealed turnover values of 0.30 to 0.73 year^–1^. These values appear to be lower than the mean values (0.76–1.40 year^–1^) determined for Scots pine in European forests (Brunner et al., 2013), and reach lower values than those of other tree species (0.48 ± 0.52 year^–1^; Finer et al., 2011), both estimated by the ingrowth method. Yuan and Chen (2010) analyzed fine roots worldwide, obtaining a mean turnover rate of 0.61 ± 0.17 year^–1^ for pines. While our values are still in the range covered by these studies, Finer et al. (2011) stated that the ingrowth core method usually yields lower turnover values than those obtained by the sequential coring or minirhizotron method, most likely because of a shorter exposure (<2 year). Keeping in mind the turnover values of 0.5 to 1.0 year^–1^ obtained by Solly et al. (2018), our values are in a similar range, with the exception of 2016, which was remarkably low at 0.3 year^–1^.

Comparing the two methods, soil coring and ingrowth cores, has been done previously (e.g., Persson, 1983; Neill, 1992; Makkonen and Helmisaari, 1999), but mainly to compare dynamic fine-root traits and not to compare morphological or architectural traits. Our data shows that the experimental treatment in the Pfynwald forest had a similar effect across methods, although absolute values significantly differed between methods for most traits. Hendricks et al. (2006) concluded that ingrowth core estimates are in general comparable with soil core estimates, although ingrowth cores may underestimate root production in water-limited environments. In our study, we observed a similar trend regarding fine-root biomass, where values obtained from ingrowth cores were about one-third of those from soil cores.

Drought is known to affect both fine-root traits, such as the dynamic traits of biomass and length, and physiological and molecular traits (Gessler et al., 2005; Brunner et al., 2015; Volkmann et al., 2016; Polle et al., 2019). The production of the plant hormone abscisic acid (ABA) is strongly enhanced, influencing root growth, as well as aquaporins and proline synthesis, which help to regulate water uptake and contribute to osmotic adjustment. Hydraulic conduits and suberin are increasingly produced to better handle water transport and water loss, respectively (Fonti and Jansen, 2012; Barberon et al., 2016). Morphological and architectural traits, in contrast, do not appear to be strongly affected by water limitation. Weemstra et al. (2017) investigated fine-root trait plasticity in European beech and Norway spruce in two contrasting soils (clay vs. sand) and observed that dynamic traits, such as biomass and length, were variable, but that morphological traits such as diameter, SRL and RTD were not. It is indeed hypothesized that dynamic traits follow the resource economics spectrum (Grime, 1977; Bardgett et al., 2014; Reich, 2014), and are thus influenced by soil resource availability (i.e., water and nutrients). This study has come to the same conclusion, that while biomass and length were found to be positively affected by the irrigation treatment – and thus by soil water availability – diameter, SRL, and RTD remained unaffected.

In general, roots can show considerable trait plasticity (Callaway et al., 2003). A recent meta-analysis of the effects of precipitation changes on fine-root traits at a global scale concluded that the responses of fine-root biomass, production, decomposition, and morphology to precipitation can be either positive or negative (Zhang et al., 2019). Joslin et al. (2000) previously reviewed the dynamics of fine-root biomass in long-term stand-level irrigation experiments (2–4 year) with no conclusive results. Particularly with European beech, results were ambiguous, as decreases in precipitation resulted in more fine-root biomass in some cases (e.g., Hertel et al., 2013) and less in others (e.g., Meier and Leuschner, 2008). Hommel et al. (2016) showed that the allocation of assimilates to roots in European beech and Norway maple (*Acer platanoides*) increased under mild, but strongly decreased under more intensive drought, suggesting that drought regimes may impact fine-root biomass production. While there are indications that water sensing by the root cap might stimulate root growth along moisture gradients allowing plants to forage water (Eapen et al., 2005; Dietrich, 2018) strong drought will impair root metabolic activity (Chaves et al., 2003; Beck et al., 2007). Hagedorn et al. (2016) showed that the allocation of assimilates to the roots under drought is driven by this root metabolic activity rather than by an impairment of phloem transport. In coordination with impaired root activity and growth, supply with new carbon is ceased. Some studies with conifers have shown biomass increases (e.g., Gower et al., 1992; De Visser et al., 1994), while others have not (e.g., Bredemeier et al., 1998). Bakker et al. (2009) also conducted a long-term irrigation experiment on 13-year-old maritime pine (*Pinus pinaster*), and overall found no effects on fine-root length, SRL, or ramification after 7 years of treatment. However, in the latter study, we must assume that non-irrigated control plants did not suffer from a lack of water, because the mean annual precipitation of the study site is approximately 950 mm. This means that plants under both treatments, control and irrigation, were most likely not water-limited. In contrast, Konopka and Lukac (2013), conducting a rain-shelter experiment in a 90-year-old Norway spruce (*Picea abies*) stand, found that water shortage reduced fine-root biomass and increased necromass, which supports the hypothesis that biomass is expected to be affected only when water is limited. Gaul et al. (2008), in a rain-shelter experiment with Norway spruce, showed that experimental drought did not result in significant changes in fine-root biomass during a six-week treatment period. We can assume here, however, that 6 weeks is too short a period to observe acclimation of tree root biomass.

Investigating the fine roots of Scots pine across a temperature and latitudinal gradient in Europe, Zadworny et al. (2017) observed that the root diameter of absorptive roots (defined by the absence of a suberized cell layer) was variable, with the diameter decreasing as the mean annual temperature (MAT) increased (0.4 mm at −2°C; 0.15 mm at 8°C). In contrast, SRL and the carbon-to-nitrogen ratio of absorptive roots were not dependent on the MAT. The average root age of transport roots (seventh to ninth root order), however, was variable with the MAT, with root age decreasing with increasing MAT (4 year at −2°C; 1.4 year at 8°C). Other studies with Scots pine, especially on precipitation gradients, are, unfortunately, lacking. Nevertheless, Zadworny et al. (2017) study provide insight into which Scots pine root traits are potentially plastic and which are not.

Zadworny et al. (2017) proposed that there are two major strategic paths that plants use. Either plants produce thin roots (with a high SRL), which grow quickly and are able to enhance water and nutrient acquisition, or they produce thick roots (with low SRL and high RTD), which grow slowly and are able to live longer, store resources, and are better protected against environmental hazards. In fact, having larger root diameters may be a more effective strategy to enhance resource acquisition when the environment is nutrient-limited (Zadworny et al., 2017). In our study, however, larger diameters and altered SRL or RTD were not obvious due to the irrigation treatment. Even though we investigated these traits with two different methods (soil coring and ingrowth cores), no significant shift in either direction was observed.

In a recent analysis of root traits, Liese et al. (2017) demonstrated that the branching pattern of roots enables a plastic response to a changing environment. Species with high branching intensity are able to rapidly and extensively proliferate into resource-rich patches. Consequently, fine-root branching, including tips and forks, and not necessarily the diameter, reflects the environment. It should be noted, however, that the goal of that study was to investigate inter-species and not intra-species variation. A better understanding could potentially be gained from the new global Fine-Root Ecology Database FRED, introduced by Iversen et al. (2017). In their review, the authors ask the question “How do root traits vary along environmental gradients?” and refer to the studies of Larson and Funk (2016) and Zadworny et al. (2016) in relation to phenotypic responses to environmental change, although the former study did not investigate trees. Overall, it appears that soil factors have a strong effect on intra-species plasticity, as Valverde-Barrantes et al. (2013) pointed out when investigating 14 angiosperm tree species. Biomass, diameter, SRL, and RTD, were found to be significantly correlated with soil fertility, fractals (=tip abundance) with soil moisture, and length with soil carbon.

The use of single-pool and diameter-based classification of fine-roots, defined here as <2 mm diameter roots, has limitations. Functional aspects of the fine roots can hardly be described with this classification system. To overcome these limitations, McCormack et al. (2015) proposed an alternative approach using an order-based classification that allows more standardized comparisons of root traits, and a functional classification that allows for the distinction between absorption and transport functions. Absorptive fine roots in their concept correspond to the most distal roots, which are mainly involved in acquisition and uptake, while transport fine roots in the branching hierarchy are higher ordered and serve mainly structural and transport functions (McCormack et al., 2015). Such a new approach would allow for more consistent and accurate comparisons of root traits as well as comparisons between functionally similar roots in the future.

## Conclusion

Our study demonstrates that the fine-root biomass of Scots pine in the topsoil of a water-limited site increases when water is added to the soil. Other studies with broader purposes (meta-analyses and reviews) have shown, in contrast, that the reactions of fine roots can be ambiguous and divergent, with increasing soil moisture leading either to an increase in fine-root biomass, a decrease, or no reaction at all. In these cases, the experimental design or the climatic gradient under study played a crucial role, and factors such as the length of the experiment, soil type, or method used to measure the fine roots influenced the results. Even though we observed in our study an increase in the fine-root biomass in the topsoil, we did not record root acclimation at deeper soil horizons. Given that the Pfynwald is located on a former alluvial fan composed of large rocks, a change in fine-root biomass at deeper levels is likely, although its measurement is not feasible. Since we observed a positive change solely in terms of fine-root biomass and length, and not in other morphological or architectural traits, such as diameter, SRL, RTD, tips, or forks, we can assume that the majority of root traits are not plastic. As Rewald et al. (2014) and Pierret et al. (2016) correctly pointed out, it is complicated to study whole root systems, and intra-root variability occurs with respect to dynamic, morphological, and architectural traits. As observed in our study, some traits undergo a change only after several years of treatment, suggesting that more research on long-term experiments in natural forests is needed. To understand why certain fine-root traits do adjust to soil environment changes and some do not, requires “more attention to the underlying drivers of fine-root mass and morphology, and to other mechanisms that are involved in soil resource uptake” (Weemstra et al., 2017). The same authors speculate that plasticity in fine-root biomass may be even more important than morphological plasticity. Further, it must be taken into consideration that the plasticity of certain fine-root traits might be species-specific, or dependent on the evolutionary status (gymnosperms, angiosperms, monocots, dicots) or mycorrhizal status (ectomycorrhizal, arbuscular mycorrhizal, ericoid, non-mycorrhizal) of the trees in question (Comas et al., 2014; Valverde-Barrantes et al., 2017). In conclusion, more studies are needed to investigate the main drivers and patterns of root-trait plasticity to finally link those to overall tree performance. Under future global change scenarios, it will be highly relevant to know which fine-root traits are susceptible to environmental changes, whether they be water, temperature, pH, or nutrients, and which are not.

## Author Contributions

IB and CH dealt with the 2005 ingrowth cores and the soil coring from 2003–2016 and analyzed the data with WinRHIZO Software. LG and AG dealt with the 2016 ingrowth cores. All authors contributed to the writing of the manuscript.

## Conflict of Interest Statement

The authors declare that the research was conducted in the absence of any commercial or financial relationships that could be construed as a potential conflict of interest.

## Footnotes

1 http://roots.ornl.gov

2 https://www.socscistatistics.com/tests/kolmogorov/Default.aspx
